# Galen vein malformation—intrauterine treatment

**DOI:** 10.1007/s00381-026-07261-5

**Published:** 2026-04-25

**Authors:** Sergio Cavalheiro, Marcos Devanir Silva da Costa, Paloam Cardoso Nôvo, Michel Eli Frudit, Fernando Seiji Suzuki, Maurício Mendes Barbosa, Patrícia Alessandra Dastoli, Stéphanno Gomes Pereira Sarmento, Ítalo Capraro Suriano, Antônio Fernandes Moron

**Affiliations:** 1 Department Neurology and Neurosurgery, Universidade Federal de Sao Paulo, Rua Napoleão de Barros, 715, 6 Floor, Sao Paulo, SP 04024-002 Brazil; 2 Fetal Medicine Division, Hospital e Maternidade Santa Joana, São Paulo-SP, Brazil

**Keywords:** Fetal vein of Galen malformation, In utero embolization, Fetal neurointervention, Prenatal diagnosis and management

## Abstract

**Introduction:**

Vein of Galen malformation (VOGM) is a rare and severe congenital arteriovenous malformation. Its therapeutic results are complex and often catastrophic, making its treatment a true challenge.

**Historical background:**

VOGM is characterized by the persistence of direct arteriovenous communications between the choroidal feeding arteries and the embryonic vein, known as the median prosencephalic vein of Markowski.

**Clinical presentation, diagnosis, and pathophysiology:**

The malformation develops between the 8th and 11th weeks of gestation. Diagnostic suspicion is usually made through fetal ultrasound after the 18th week of gestation or later. Fetal MRI complements the diagnosis.

**Management, prognosis, and outcomes:**

There are two groups of patients with VOGM: severe cases, with persistent high-flow arteriovenous shunts leading to multiorgan failure, and mild cases with a lower mortality rate and a more favorable outcome. The use of vascular parameters, based on the diameter of the falcine sinus measured by fetal MRI, can serve as a predictor of neonatal outcome and therapeutic choice.

**Case report:**

A 33-year-old primigravida presented with a fetus diagnosed with VOGM by fetal ultrasound at 32 weeks of gestation. An aneurysmal dilation of the Galen vein was observed, with a transverse falcine sinus measuring 10.5 mm. Aneurysm dilatation dimensions: 25 × 21 × 20 mm. Fetal MRI confirmed a falcine sinus width of 10.5 mm, indicating a 96% chance of poor outcome. Fetal embolization of the VOGM was performed, followed by postnatal treatment.

**Conclusions:**

In this article, we review the main principles of VOGM management and present a case treated during pregnancy, illustrating a potential therapeutic strategy. We suggest that fetal embolization of VOGM, when appropriately indicated, may represent an emerging approach in the management of this severe condition.

## Introduction

Vein of Galen malformations (VOGM) are considered congenital arteriovenous anomalies and account for approximately one-third of all pediatric vascular malformations [1, 2].

VOGM is a severe congenital vascular anomaly that poses substantial diagnostic and therapeutic challenges and is associated with significant morbidity and mortality, representing a challenge for neurosurgeons from initial clinical suspicion through diagnosis, treatment, follow-up, and prognosis [1, 2]., representing a significant challenge for neurosurgeons from initial clinical suspicion through diagnosis, treatment, follow-up, and prognosis [1, 2].

VOGM accounts for less than 1% of all intracranial vascular malformations but represents approximately 30% of those diagnosed in the neonatal period. Its incidence ranges from 1 in 25,000 to 1 in 50,000 live births. Clinically, it most commonly presents in the neonatal period and is frequently associated with high-output cardiac failure and significant morbidity and mortality, particularly in cases diagnosed early with more severe hemodynamic compromise [1, 2, 5, 6].

The development of endovascular techniques, particularly embolization, has significantly reduced morbidity and mortality rates in patients with VOGM. However, several studies still report high mortality and neurocognitive morbidity among survivors [1, 3–5].

Currently, fetal embolization may be considered a potential treatment option when specific selection criteria are met. The aim of this article is to report a case of vein of Galen malformation successfully treated using an intrauterine approach and to explore the potential role of this strategy as an emerging therapeutic option.

## Historical background

### Embryology and definition

VOGM is characterized by the persistence of direct arteriovenous communications between the choroidal feeding arteries and an embryonic vein known as the median prosencephalic vein of Markowski [1, 6–8]. Due to the lack of regression of this vein around the 11th week of gestation, and the continuous direct arterial flow, the Markowski vein becomes dilated, forming a midline varix that is typically described as an aneurysmal dilatation of the vein of Galen [4].

However, the embryological background of this pathology must be understood. Before vascular development, venous drainage is exclusively meningeal and choroidal. During the 5th and 6th weeks of gestation, the vascular system is confined to the primitive meninx. This pericerebral network divides into a deep layer (future pial layer) covering the brain surface and a superficial layer (future dural layer).

The deep layer forms a simple capillary plexus, while in the superficial layer, the vascular channels become continuous and connect to the paired aortas and cardinal veins, giving rise to the initial arterial and venous trunks [9].

Between the 6th and 8th weeks, the choroidal tela differentiates and, to better support the developing brain tissue, forms the intraventricular choroid plexus. Consequently, specific feeding and draining channels emerge. The feeding arteries are the anterior cerebral artery (ACA), anterior choroidal artery (ACHA), and posterior choroidal artery (PCHA).

The principal collector of the superior choroidal veins is a single median dorsal vein, known as the median prosencephalic vein, first described by Markowski in 1911 [9, 10].

The Markowski vein serves as the specific drainage vein of the choroidal plexuses, appearing as soon as the choroidal arteries are identified (around the 6th week). By approximately the 11th week, this vein regresses and disappears at the end of the choroidal stage, being replaced by the vein of Galen as subependymal drainage develops following the maturation of intrinsic vascularization in the marginal zones [9].

The first point to clarify is that the term “aneurysm of the vein of Galen,” although widely used, is embryologically inaccurate. Rather than a simple aneurysm, it represents a true arteriovenous malformation (AVM). The malformation is supplied anteriorly by the ACA, ACHA, and PCHA, and posteriorly by the dorsal mesencephalic arterial plexus [9].

Furthermore, there is always a single venous pouch, which can be identified not as the vein of Galen itself, but rather as the dorsal prosencephalic vein (Markowski vein). Embryologically, this structure is not identified before the 8th week or after the 11th week of gestation. This understanding has greatly improved our comprehension of the pathophysiology of VOGM [9].

## Clinical presentation, diagnosis, and pathophysiology

Chronologically, the malformation arises during the brief developmental window in which the meninges and choroid plexuses constitute the main structures supplying the neural tissue. This period extends approximately from the 8th to the 11th week of gestation [9]. The event responsible for the formation of the fistula is believed to occur around the 8th week, and the resulting high-flow shunt prevents the regression of the embryonic vein [9].

**Fig. 1:**
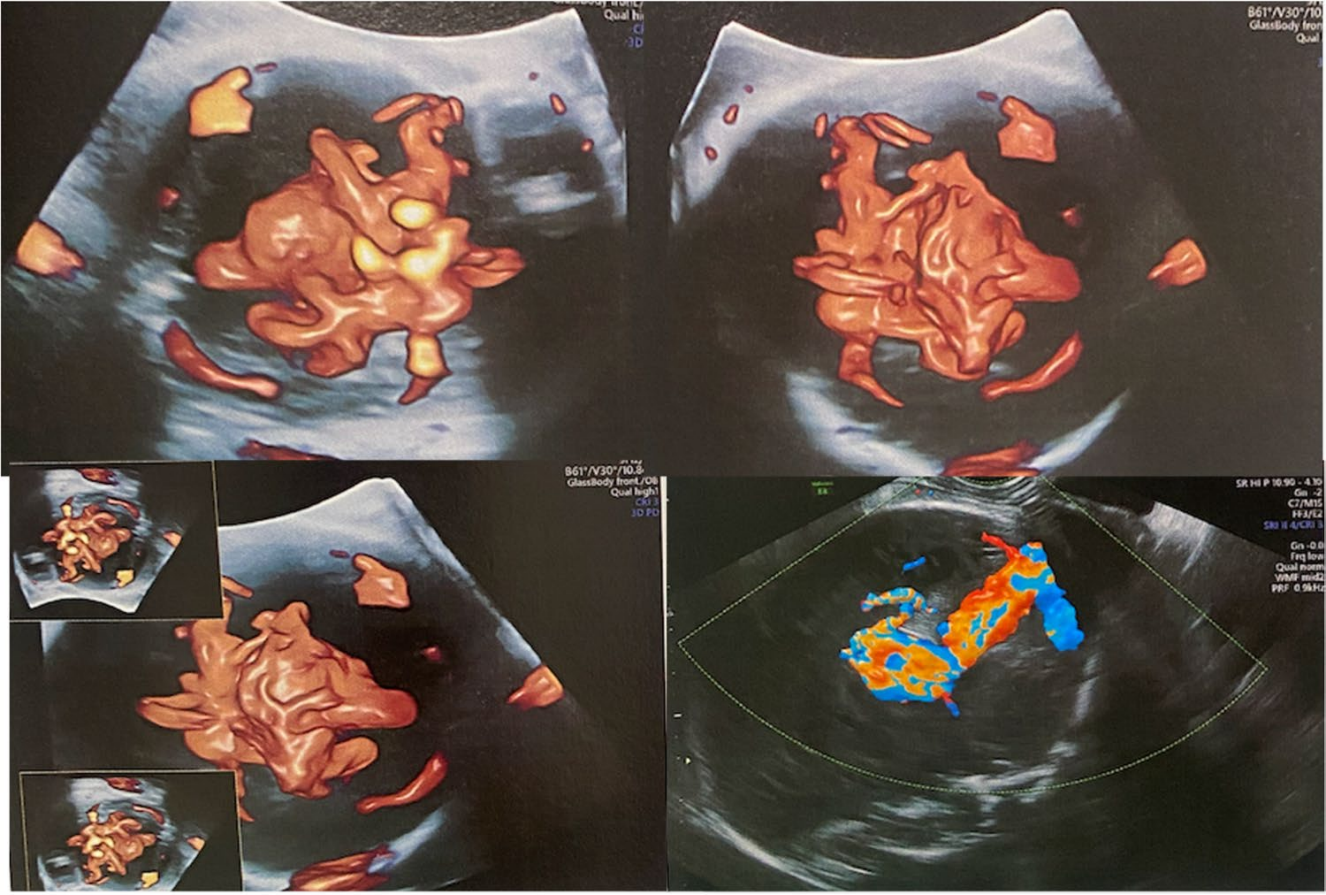
Obstetric Doppler ultrasonography demonstrating an aneurysmal dilation of the vein of Galen (present-case images)

In clinical practice, the prenatal diagnosis is usually made during the third trimester, with color Doppler ultrasonography demonstrating turbulent arterial and venous flow within a hypoechogenic midline structure located posterior to the third ventricle. The anatomical configuration of VOGM—characterized by a dilated, persistent prosencephalic (Markowski) venous varix in the midline, supplied by choroidal arteries—is pathognomonic of the diagnosis (Fig. 1).

**Fig. 2:**
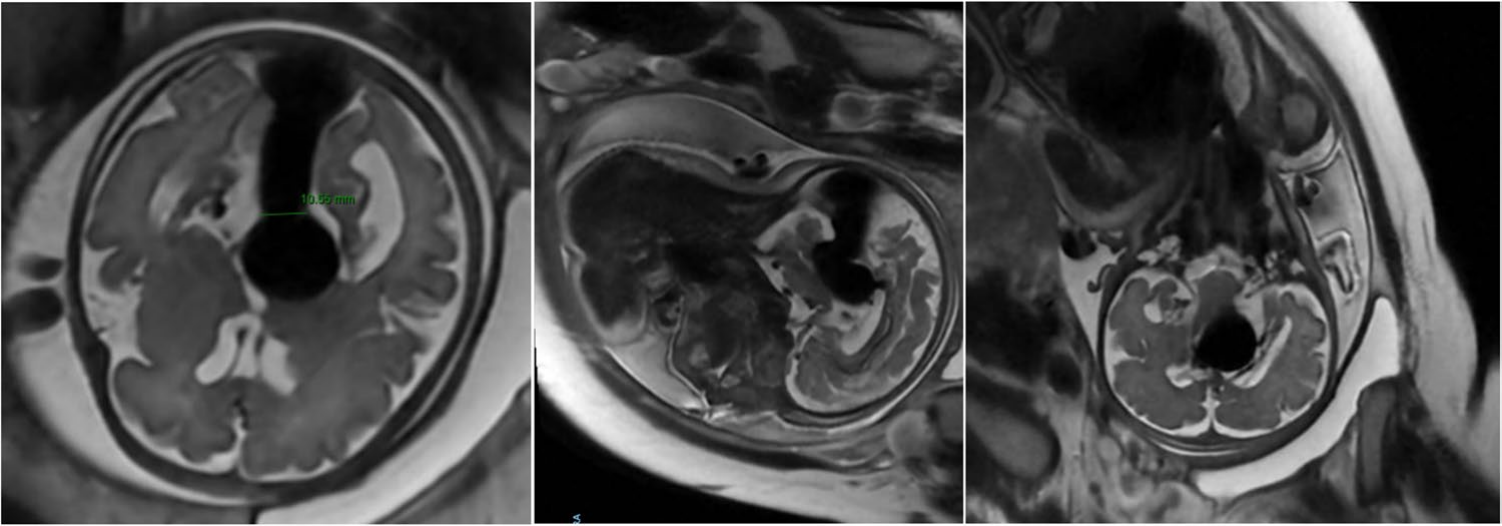
Fetal MRI of the clinical case demonstrating a vein of Galen malformation (VOGM) (Images from the present case)

Fetal MRI is essential to confirm the diagnosis of VOGM, detect associated brain abnormalities, and rule out differential diagnoses, including arachnoid, porencephalic, or choroid plexus cysts, pineal tumors, choroid plexus papilloma, and even intracranial hematomas [3] (Fig. 2).

VOGMs are known causes of high-output cardiac failure in the fetus and/or neonate, often accompanied by cardiac dilation [11–13], as well as pulmonary hypertension and, in extreme cases, multisystem organ failure [11, 14]. Pulmonary hypertension is presumed to result from increased pulmonary blood flow [11].

With the advent of fetal imaging techniques, VOGMs have been shown to impose a significant hemodynamic burden on the fetus [11]. The increase in cardiac output in this and other high-output states leads to worsening of the cardiovascular profile score, a marker of cardiac performance [15].

It is reasonable to assume that VOGMs are associated with dilated cardiac structures, predominantly affecting the right heart. Markers of right ventricular (RV) dilation (such as increased RV z-score), RV dysfunction, and cardiomegaly may even predict disease severity [11].

Case reports and small series suggest that the cardiac effects of VOGM, particularly hydrops fetalis, cardiomegaly, dilated systemic veins, retrograde aortic flow, tricuspid regurgitation, and pericardial effusion—are associated with poor outcomes. Godfrey et al. [11] demonstrated that increasing shunt volume leads to a greater hemodynamic burden on the right side of the heart, increasing the risk of postnatal heart failure, pulmonary hypertension, and reduced survival, as well as cerebral injury secondary to vascular steal phenomena.

More specifically, Paladini et al. [17] identified tricuspid regurgitation as a significant predictor of brain injury. In addition, the presence of tricuspid regurgitation associated with a VOGM varix volume greater than 20 mL was correlated with severe neurological impairment, death, or late termination of pregnancy. This association was robust, with 20 of 29 cases with volumes > 20 mL resulting in poor outcomes.

These findings suggest that quantitative parameters, such as the presence of tricuspid regurgitation and varix volume greater than 20 mL, may serve as important markers of disease severity and predictors of worse prognosis [17].

Therefore, VOGM often presents to the fetal cardiologist with findings of right-heart enlargement on obstetric ultrasound. It is the role of the fetal cardiologist to determine the etiology, differentiating primary congenital heart disease from extracardiac anomalies. For instance, a malformation located in the upper body that drains into the superior vena cava and preferentially into the right ventricle is more likely to cause disproportionate right-heart enlargement compared with the left [11].

## Management, prognosis, and outcomes

### Prediction of prognosis and a proposal for fetal treatment

In severe cases, the persistent arteriovenous shunt of VOGM can lead to multiorgan failure, progressive destruction of the hemispheric white matter, and cerebral parenchymal injury [1, 16–18].

However, there is a subset of patients who evolve with lower mortality rates and favorable outcomes, both in intensive and neurosurgical care, allowing for elective treatment within the first months of life.

Despite advances in prenatal detection and the development of endovascular embolization as a treatment option, predicting outcomes in infants with VOGM—particularly neurological outcomes—remains a challenge.

In a study of 49 cases, the prognostic value of various prenatal variables was evaluated to identify potential indicators of poor outcomes in terms of mortality and cerebral disability [17].

Severe brain lesions evident on ultrasound or MRI were, by definition, considered associated with unfavorable outcomes in all cases. Moreover, the study identified tricuspid regurgitation as a predictor of cerebral injury, and both regurgitation and a varix volume greater than 20 mL were associated with severe neurological impairment, death, or late-pregnancy termination due to severe fetal brain abnormalities. However, the need for intervention was not addressed in this analysis, and these findings have not been replicated [17].

In an effort to distinguish between the two prognostic groups—patients with favorable and unfavorable outcomes—the use of selected vascular parameters on fetal MRI, such as the narrowest point of venous constriction where the malformation drains into the systemic circulation, has been proposed as a predictor of neonatal outcome.

In the study by Arko et al., 2020 [1], 32 patients were stratified into two groups: high-risk neonates, requiring urgent neonatal intervention or resulting in neonatal death, and clinically stable infants, who did not require immediate treatment. Based on these two cohorts, vascular measurements were obtained from fetal MRI to identify reliable predictors of mortality.

The maximum mediolateral diameter and cross-sectional area at the narrowest point of the straight or falcine sinus were found to be the most predictive parameters of clinical risk. A larger mediolateral diameter or cross-sectional area at the shortest segment of the straight or falcine sinus correlated with a significantly higher risk of adverse outcome, establishing this as a predictive marker of mortality and need for neonatal intervention [1].

**Fig. 3:**
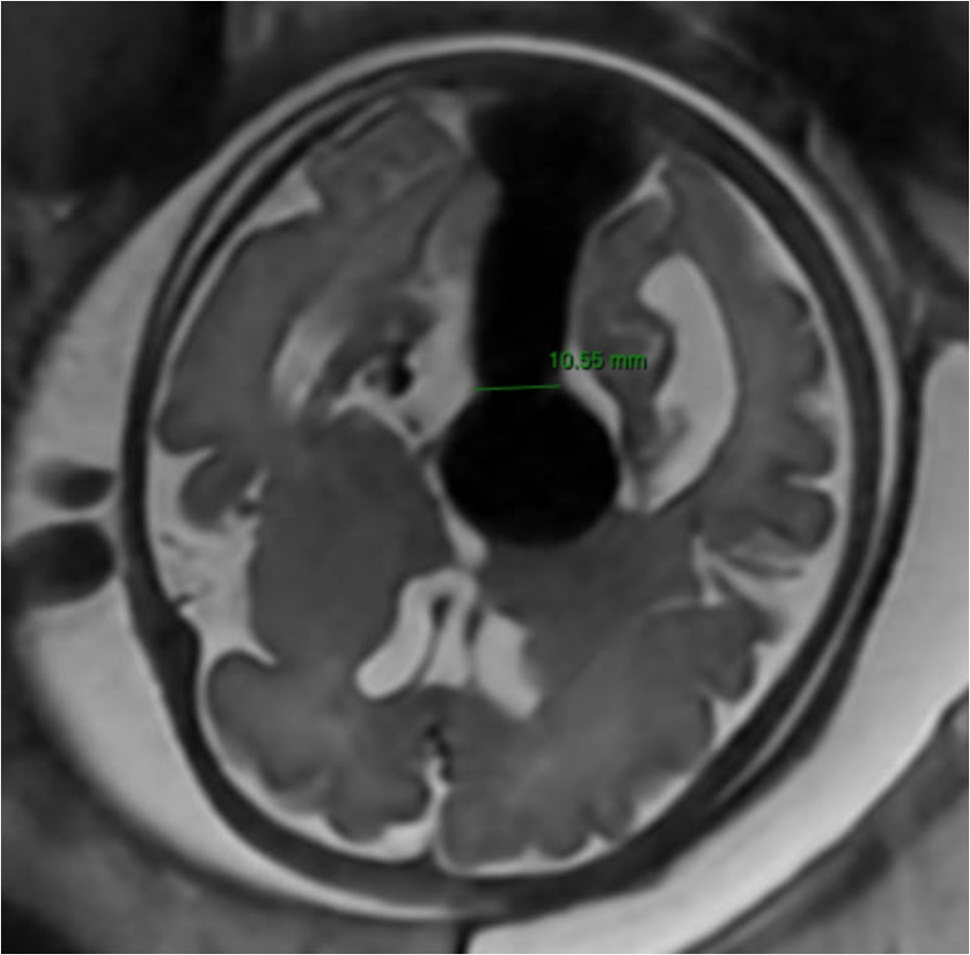
Fetal MRI of the clinical case showing measurement of the falcine sinus at its narrowest point, measuring 10.5 mm

Using a threshold of 8 mm for the mediolateral width of the falcine sinus, Arko et al. [1] reported an approximately 88% probability of classification into the high-risk neonatal group (Fig. 3).

This specific parameter is consistent with the pathophysiology of the disease, since the point of greatest constriction of the venous sinus draining the prosencephalic varix represents a definitive limiting site for the return of the malformation’s blood flow to the systemic circulation [4].

In another retrospective study involving 59 patients diagnosed with VOGM by MRI, with a median gestational age of 34 weeks, the mediolateral diameter of the falcine sinus had a median value of 6 mm in the cohort and was found to predict mortality, right ventricular systolic dysfunction at discharge (*P* = 0.02), cerebral parenchymal abnormalities at birth (*P* = 0.02) and at discharge (*P* = 0.02), ventriculomegaly at birth (*P* = 0.03), and developmental delay at 1 month (*P* = 0.001), 6 months (*P* = 0.001), and 12 months (*P* = 0.002) [16].

The study further expanded the application of falcine sinus measurement to predict broader clinical effects and demonstrated that it serves as a robust predictor for a range of important clinical, radiological, and developmental outcomes [16].

To determine the safety of fetal embolization for patients with VOGM, and secondarily to evaluate its efficacy, Pokmeng See et al. [4] recently published a protocol for a clinical safety and feasibility trial. It is a prospective, single-arm, non-randomized, interventional study employing a single fetal embolization procedure followed by periodic evaluations.

Inclusion criteria include pregnant patients with fetuses diagnosed with VOGM in which the mediolateral width of the venous drainage sinus (falcine or straight sinus) measures 8 mm or more at its narrowest point on fetal MRI, maternal age ≥ 18 years, and gestational age between 23 weeks and term [4].

Exclusion criteria include extensive fetal cerebral parenchymal injury or gliosis involving > 10% of supratentorial brain volume, irreversible non-cerebral fetal organ injury (e.g., hydrops fetalis as a manifestation of heart failure), fetuses with VOGM in which the draining straight or falcine sinus measures < 8 mm on fetal MRI, and other specific exclusion parameters defined in the study protocol [4].

The clinical and imaging parameters used to identify candidates for fetal embolization include evidence of a high-flow arteriovenous shunt on fetal imaging, as well as signs of progressive hemodynamic compromise, such as cardiomegaly, tricuspid regurgitation, or hydrops fetalis. Imaging findings suggestive of worsening cerebral venous hypertension or progressive ventriculomegaly were also considered [1, 11, 17, 20].

Additionally, the mediolateral diameter or cross-sectional width of the straight/falcine sinus was considered, with values greater than 8 mm being associated with a higher risk of adverse outcomes, as previously described in the literature. However, we acknowledge that this threshold may be overly restrictive and could potentially exclude fetuses who might still benefit from intrauterine intervention despite measurements below this value [1].

We also emphasize that the presence of severe and irreversible brain injury represents a contraindication to intervention. Therefore, fetal embolization is currently reserved for selected cases evaluated within a multidisciplinary setting [1, 4, 20].

**Fig. 4:**
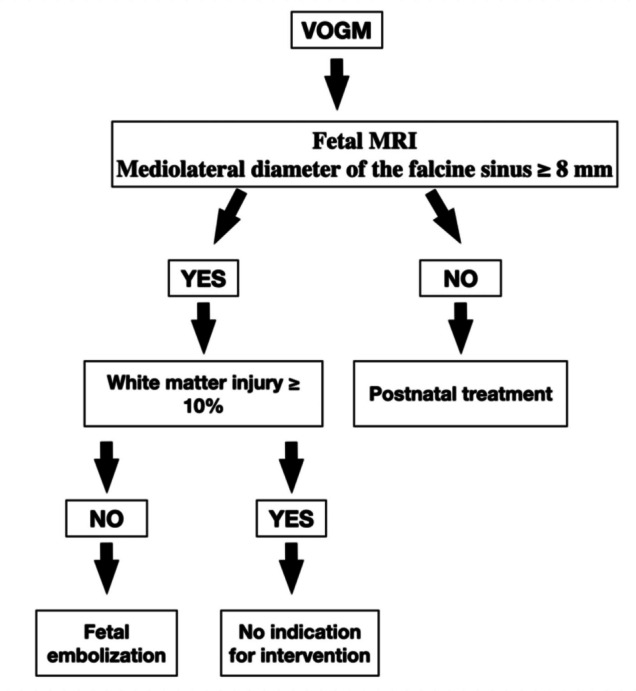
Simplified schematic of the proposed fetal treatment approach for VOGM, as applied in the reported case. Based on the studies by Pokmeng See et al. [4] and Arko et al. [1]

Based on these findings and the current published literature, we propose a simplified flowchart to guide fetal treatment strategies for patients with VOGM (Fig. 4).

### Fetal embolization

The high mortality and morbidity associated with microsurgical treatment—exceeding 90% mortality in some series—led to the abandonment of microsurgery and the development of endovascular techniques, which since the 1980 s have become the first-line treatment for VOGM, marking the end of the microsurgical era [19–21].

The outcomes in the embolization era are undeniably better; however, concerns remain regarding case selection, as embolized cases typically represent patients who survived several months postnatally [19–21].

Nevertheless, there exists a subset of patients with poor prognosis who die in utero or shortly after birth. This has prompted investigations into prognostic factors to determine whether prenatal treatment could be offered to selected patients.

From a pathophysiological standpoint, the low resistance within the malformation induces high-flow shunting, leading to cerebral hypoperfusion and cardiopulmonary stress. In utero, the placenta—also a low-resistance vascular bed—can provide hemodynamic compensation and fetal protection [22].

This suggests that VOGM can remain compensated for a prolonged period, and decompensation may be related to the rapid increase in cortical vascularization during the third trimester, as well as factors such as closure of the ductus arteriosus and loss of placental reserve immediately after birth [9, 22].

Based on this reasoning, it is plausible to hypothesize that the clinical outcome of patients with VOGM could be improved if intervention were performed before the onset of acute postnatal cardiovascular and cerebrovascular stress.

The first reported case of fetal embolization for VOGM was presented at the 2014 International Congress of Ultrasound in Obstetrics and Gynecology by Mendez et al. [23]. The fetus, at 30.4 weeks’ gestation, presented with VOGM and hydrops fetalis. The procedure was performed at 31 weeks under ultrasound guidance: an 18-gauge percutaneous transthoracic needle was introduced into the venous lumen, followed by advancement of a 1.9-F microcatheter through the needle and positioning at the aneurysmal center, where eleven platinum coils were sequentially deployed into the vein of Galen, achieving complete occlusion [23].

After the fetal procedure, there was progressive reduction of flow within the vein of Galen and complete resolution of hydrops within one week. The infant was born alive at 35.4 weeks of gestation, without signs of heart failure. However, neonatal death occurred on the 10th postnatal day, secondary to unexplained renal failure [23].

Another case of fetal embolization, registered on ClinicalTrials.gov and conducted under FDA supervision within an Institutional Review Board-approved protocol, utilized a percutaneous, ultrasound-guided transcranial embolization technique and was published by Orbach et al. (2023) [22].

The procedure was performed at 34 weeks gestation. An immediate and marked reduction in variceal flow was observed on intraoperative ultrasound. After embolization, fetal echocardiography demonstrated a 43% reduction in total cardiac output. Fetal MRI showed decreased caliber of the prosencephalic varix and reduction in falcine sinus width, with no evidence of subdural, subarachnoid, or intraventricular hemorrhage or infarction [22].

The same study published its first results earlier this year, reporting that among seven included cases, five underwent successful in utero embolization [24].

Our reported case of fetal embolization was technically successful and represents the fourth documented case in the current literature. We therefore recognize this as a new frontier to be explored in the management of this challenging disease [25]. (Table 1).

**Table 1:** Chronological order of published cases of in utero embolization for VOGM

Author	IGd	IGe	IGp	LMLS	Coils	Volume	Follow-up	Outcome
Martínez et al. (2014) [23]	30, 4	31	35, 4	-	11	-	10 days	Death
Orbach et al. (2023) [22]	30, 5	34, 2	34, 4	12, 9 mm	23	14.600 mm^3^	21 days	Improvement
Naggara et al. (2024)	31, 4	33	38, 2	10, 4 mm	10	-	30 days	Improvement
Present case (2024)	32	33, 2	36, 3	10, 5 mm	15	10.500 mm^3^	60 days	Improvement
Orbach et al. (2025) [24]	-	35, 6	-	10, 3 mm	-	10.300 mm^3^	6 months	5 cases/2 deaths

Hyphens (-) denote “not reported”

*IGd* gestational age at diagnosis, *IGe* gestational age at embolization, *IGp* gestational age at delivery, *LMLS* mediolateral width of the sinus

## Case report

**Fig. 5:**
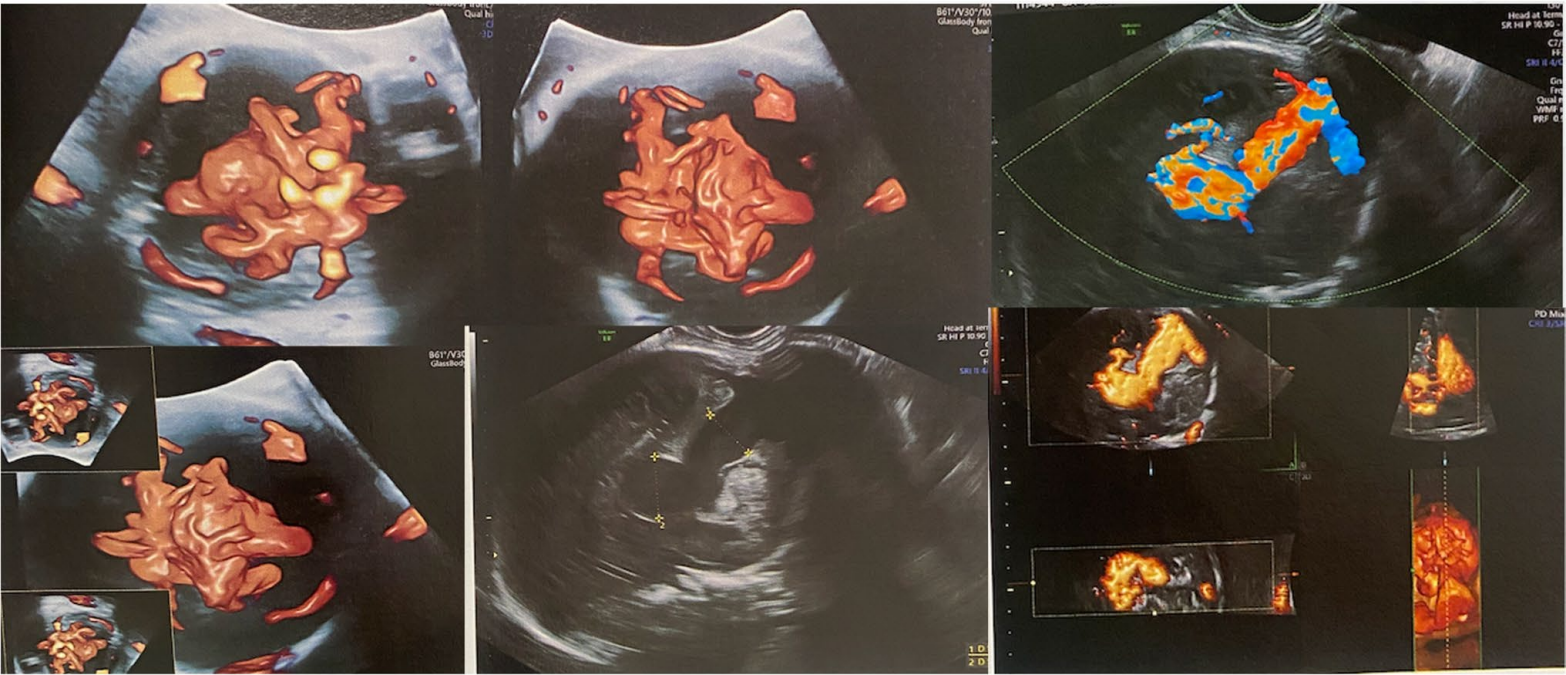
Obstetric Doppler ultrasonography at 32 + 5 weeks of gestation demonstrating a large Vein of Galen Malformation, measuring 10,500 mm^3^ in volume and a falcine sinus width of 10.5 mm

A 33-year-old healthy primigravida presented with a fetus at 32 + 5 weeks of gestation showing cardiac failure associated with moderate tricuspid valve regurgitation on fetal echocardiography. Color Doppler ultrasonography identified the presence of a vein of Galen malformation with dilatation of the falcine sinus measuring 10.5 mm. The cerebral ventricles were of normal size, and Doppler studies of the anterior and middle cerebral arteries were within normal limits. The ampulla of Galen measures 25 × 21 × 20 mm (volume = 10,500 mm^3^) (Fig. 5). The falcine sinus width measured on fetal MRI is consistent with the ultrasound findings (Fig. 3).

No evidence of cerebral ischemia or hemorrhage was detected.

Based on the falcine sinus diameter and the presence of fetal heart failure, fetal embolization of the VOGM was indicated. The procedure was performed via a percutaneous transoccipital venous approach under ultrasound guidance at 33 + 2/7 weeks of gestation. Informed consent was obtained, and the procedure was approved by the Institutional Review Board of Hospital e Maternidade Santa Joana, São Paulo, Brazil.

**Fig. 6:**
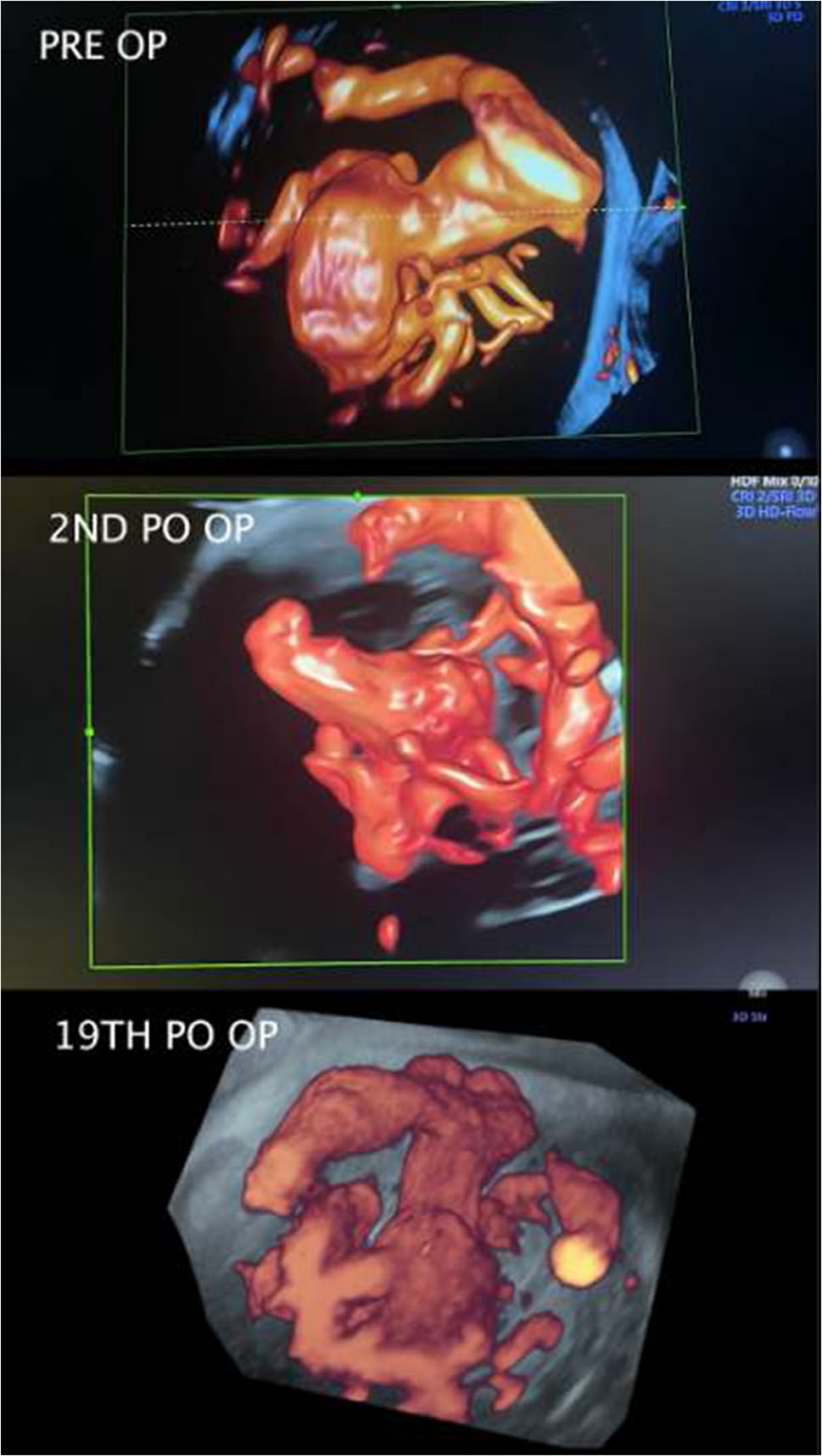
Fetal ultrasound performed on the first, second, and tenth postoperative days following in utero embolization

The ellipsoidal venous cavity (aneurysm) volume was calculated as 3769 mm^3^. Aiming for approximately 15% volumetric packing, the required number of detachable platinum coils (Target XL, Target Neurovascular, Fremont, CA, USA) was determined. The mother underwent general and spinal anesthesia, while the fetus (estimated weight 2870 g) received intramuscular anesthesia with fentanyl and pancuronium. Fetal heart rate was continuously monitored using Doppler ultrasonography throughout the procedure. After uterine relaxation, an obstetric fetal version was performed to orient the occiput anteriorly toward the maternal abdominal wall, given the posterior placental implantation. Under ultrasound guidance, an 18G Chiba needle was introduced through the abdominal and uterine walls into the amniotic cavity, directed toward the fetal occipital region. Using a rotational drilling technique with gentle forward pressure, the needle was carefully advanced through the fetal skull into the torcular Herophili. Removal of the stylet confirmed venous blood return, and the needle was connected to a pressurized saline line via a luer-lock extension tube. A microcatheter XT-27 (Target Neurovascular, Fremont, CA, USA) was advanced through a rotating hemostatic valve over a Syncro 14 microguidewire (Target Neurovascular, Fremont, CA, USA) under real-time ultrasound guidance, navigating anteriorly through the falcine sinus into the venous cavity. Because of the sharp bevel of the Chiba needle, the catheter was not withdrawn from the needle at any point during the intervention. A total of 15 detachable Target XL coils were implanted within the varix, achieving a 14.4% packing volume. A marked reduction in blood flow was immediately observed on Doppler ultrasound. The needle and catheter were withdrawn in the operating room, and the mother was transferred to the recovery area in stable condition (Fig. 6).

Echocardiographic follow-up was performed daily after the intrauterine procedure (Table 2 and Fig. 6).

**Table 2:** Summary of the fetal echocardiographic evolution

GA	CTR	AV	PV	SVC	DA	Myocardial contractility	CCO	CVI	FHR
33 1/7	0.52	1.3	1.0	7	1.65	Normal	552	6/60	133
33 2/7	0.57	1.0	1.1	5	1.5	Normal	375	6/10	121
33 3/7	0.63	1.7	1.2	7	2.36	Normal	505	6/10	121
33 4/7	0.40	1.7	1.0	6.5	1.13	Normal	500	7/10	129
33 5/7	0.38	1.2	0.89	6.1	1.48	Normal	450	7/10	125
34	0.38	1.5	0.77	6.3	1.50	Normal	425	7/10	137
35	0.38	1.3	0.95	6.5	1.20	Normal	625	7/10	128

*GA* gestational age, *CTR* cardiothoracic ratio, *AV* aortic velocity, *PV* pulmonary velocity, *SVC* superior vena cava, *DA* ductal artery (arterial duct), *CCO* combined cardiac output (normal value, 450–500 ml/kg/min), *CVI* cardiovascular index, *FHR* fetal heart rate

**Fig. 7:**
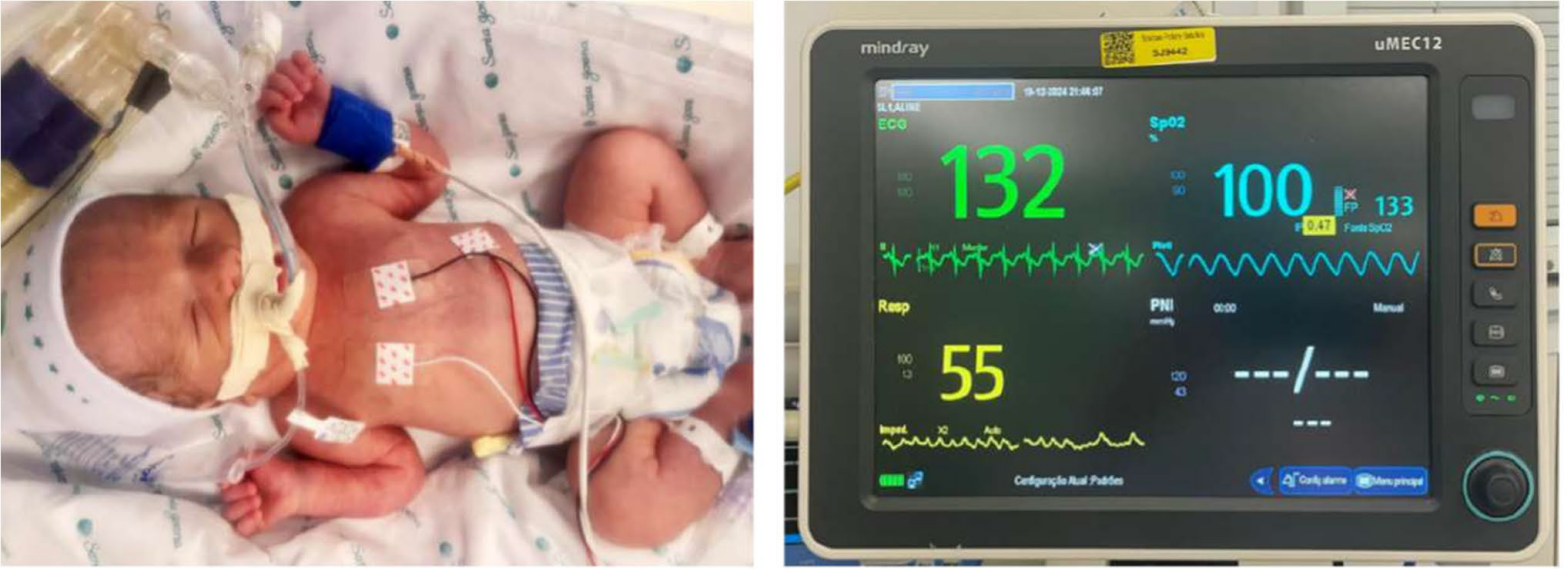
Image of the newborn in the neonatal intensive care unit (NICU) at 7 h of life

**Fig. 8:**
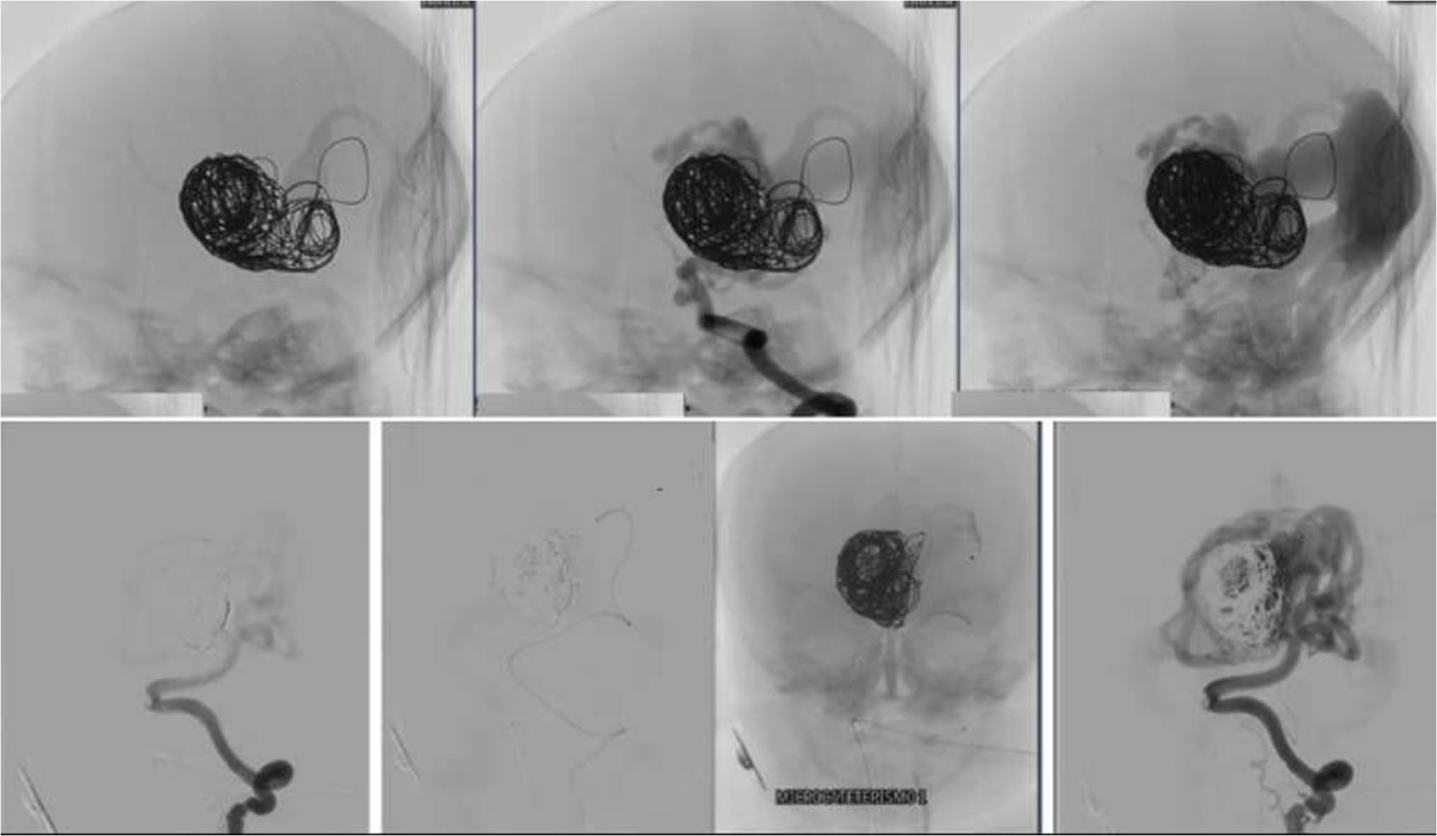
Images of the VOGM embolization procedure performed on the second day of life

A cesarean delivery was performed on December 19,2024, at 36 + 3/7 weeks of gestation. The newborn weighed 2730 g (Hadlock percentile 18.6). At birth, no spontaneous respiratory movements were observed, and endotracheal intubation was performed in the delivery room. Apgar scores were 2/4/7. Physical examination revealed a small puncture site on the occipital scalp, consistent with healing of the previous fetal intervention. The anterior fontanelle showed no palpable thrill. The neonate was edematous, tachypneic, and exhibited signs and symptoms of congestive heart failure (CHF). A second embolization session was required on the second day of life due to refractory CHF and multiorgan failure (Figs. 7 and 8). The procedure was performed via the right femoral artery. A 4 F pediatric femoral introducer was inserted under ultrasound guidance. Following catheterization of the left vertebral artery with a Tempo Aqua Diagnostic Catheter (Cordis), used as a guide catheter, a brief angiographic study of the vertebrobasilar system was performed. Multiple high-flow arteriovenous fistulas were identified along the wall of the median prosencephalic vein (vein of Galen), with a large median venous pouch partially filled by previously deployed coils. The left posterolateral and posteromedial choroidal arteries were successively catheterized using Headway Duo microcatheters (MicroVention–Terumo) and Traxcess 14 microguidewires (MicroVention–Terumo), and embolized with a 1:1 mixture of histoacryl (B. Braun) and lipiodol (Guerbet). Subsequently, the right posterolateral choroidal artery was also catheterized with a Headway Duo microcatheter and Traxcess microguidewire, and embolized using the same Histoacryl/Lipiodol 1:1 mixture. The final angiographic control demonstrated a significant reduction in arteriovenous shunting, with immediate improvement in the newborn’s hemodynamic parameters.

The objective of the fetal embolization was to achieve a substantial reduction in blood flow, rather than complete occlusion of the VOGM.

Fetal echocardiography performed on the day following the post-fetal embolization demonstrated improvement in combined cardiac output, measuring 500 mL/kg/min (normal range, 450–500 mL/kg/min), and a reduction in the feeding vessel of the ampulla of Galen aneurysm to 9 mm. Subsequent examinations showed progressive improvement, with positive A-wave in the ductus venosus (PI = 0.98), fetal heart failure score of 7/10, and combined cardiac output within the normal range. Biventricular cardiac function was preserved, and the ductus arteriosus appeared tortuous with accelerated flow.

The child is currently 15 months old (without correction for prematurity) and, during this period, required ventriculoperitoneal shunt placement. She presents with neurocognitive developmental delay, although she has not yet been evaluated using standardized scales, and continues to receive regular multidisciplinary follow-up.

## Conclusion

In this article, we report a case of in utero treatment of a vein of Galen malformation (VOGM) that resulted in neonatal survival. Further research is needed to determine the optimal technique and address existing knowledge gaps regarding management; however, we believe that fetal embolization of VOGM, when appropriately indicated, may represent a paradigm shift in the treatment of this severe condition.

## Data Availability

No datasets were generated or analysed during the current study.
